# Practical Observations on the Diseases Most Fatal to Children; with Reference to the Propriety of Treating Them as Proceeding from Irritation and Not from Inflammation

**Published:** 1845-07

**Authors:** 


ART. V.—*Practical Observations on the Diseases most fatal to Children; with reference to the propriety of treating them as proceeding from Irritation and not from Inflammation.* By P. HOOD.—*London,* 1845. 8vo, pp. 232.

No one can have been long engaged in the practice of medicine, without meeting with cases that present many of the symptoms of active inflammatory action, but which need a treatment the reverse of antiphlogistic, and in which organic changes occur very dissimilar from those which result from inflammation. Such cases are far more frequent in children than in adults, while many circumstances concur to render their discrimination in young subjects a task of exceeding difficulty. The essays of Dr. Gooch and Dr. Marshall Hall on the hydrocephaloid disease, and that of MM. Bailly and Legendre, on lobular pneumonia, show, however, that the problem may be worked with a fair prospect of success, and that its solution is in each case so important as fully to repay the most laborious investigation. When we first opened Mr. Hood's book it was with the hope of finding in it an exposition of the different characters that distinguish the inflammatory from the non-inflammatory diseases of childhood, and we trusted that even if it should not embody the results of very profound research or elaborate inquiry, it would at least present the fruits of careful observation at the bed-side of the sick, and contain much of real practical value.

We regret, however, that these expectations have been disappointed. Instead of pointing out the distinctions between inflammatory and non-inflammatory diseases, Mr. Hood has contented himself with absolutely denying the occurrence of inflammation in childhood. A few hours spent in examining the dead body would have shown him the error of such a statement. The work, however, contains no evidence that Mr. Hood ever passed a single hour in any such examination, and though professedly treating of *the diseases most fatal to children,* in no instance is there any account of the morbid appearances to which they give rise.

Having gone carefully over the book, we had thought of pointing out some of the more gross mistakes into which this neglect of morbid anatomy has led its author, but we abstain on observing that Mr. Hood addresses his lucubrations not to members of our profession only, but to "parents," and "to all persons who may be interested in the matter." Many of his statements which would otherwise have been totally inexplicable are thus fully accounted for, while we do not think it worth while to examine them ourselves since the author appeals to another tribunal than that of medical criticism. Mr. Hood, however, must have known that in appealing to any but professional men, on a subject so difficult as the investigation of the mode of treatment which may be most appropriate in the more serious diseases of children, he was bringing his cause before a jury wholly incompetent to try its merits. The fault he has committed is one which implies not mistaken judgment, but want of self-respect, want of just appreciation of the dignity of his calling, and of the dignity of truth. We regret to be compelled to speak thus about a gentleman whose work gives indications of talent quite sufficient to convince us that had his aims been higher he might have written a book that would have been honorable to himself and useful to the profession.

